# Metacognitive Therapy for Adjustment Disorder in a Patient With Newly Diagnosed Pulmonary Arterial Hypertension: A Case Report

**DOI:** 10.3389/fpsyg.2020.00143

**Published:** 2020-02-12

**Authors:** Lotta Winter, Franziska Naumann, Karen Olsson, Jan Fuge, Marius M. Hoeper, Kai G. Kahl

**Affiliations:** ^1^Department of Psychiatry, Social Psychiatry and Psychotherapy, Hannover Medical School, Hannover, Germany; ^2^Department of Pneumology, Hannover Medical School and German Centre for Lung Research (DZL), Hannover, Germany

**Keywords:** metacognitive therapy, adjustment disorder, pulmonary arterial hypertension, psychotherapy, PAH, MCT

## Abstract

Adjustment disorders (ADs) belong to the worldwide most diagnosed mental disorders and are particularly frequent in patients with an underlying physical illness. Pulmonary arterial hypertension (PAH) is a severe and disabling disease, which significantly impacts on quality of life and has high mortality rates. The authors here present the case of a young female who developed a severe adjustment disorder with both anxious and depressive symptoms after a diagnosis of PAH requiring intensive care treatment due to right heart failure. Psychosocial functioning was severely impaired, and physical health reduced. Following hemodynamic stabilization and the establishment of PAH treatment, the patient was admitted to the Department of Psychiatry, Social Psychiatry and Psychotherapy and received metacognitive therapy (MCT). AD with mixed anxiety and depressed mood was diagnosed according to DSM-V criteria. At the start of treatment, she reported significant mental distress, indicated by a total sum score of the Hospital Anxiety and Depression Scale (HADS) of 20 points. The 6-min walking distance was only 358 m before the patient was exhausted. She then was treated with MCT without further psychopharmacological drugs. After only four MCT sessions, she fully remitted from AD which was accompanied by an 11-point reduction in the HADS (to 9 points). MCT specific scores also improved (MCQ-30 sum score decreased from 77 to 35). Notably, physical capacity improved as well, documented by an improved walking distance (439 m; +22%). This is the first case of a patient with AD in the context of PAH treated with MCT. The case report suggests that MCT is a possible psychotherapeutic treatment option for AD in the context of a potentially life-threatening disease. The study design does not permit an attribution of outcome to MCT but it suggests MCT is a potentially viable and acceptable treatment option.

## Introduction

Adjustment disorder (AD) represents an abnormal stress response that is different from normal adaptive reactions (Casey, 2014). According to DSM-V, AD is characterized by: (A) emotional or behavioral symptoms in response to an identifiable stressor that (B) are of clinical significance and (C) do not meet the criteria for another mental disorder, and (D) do not represent normal bereavement. Typically, AD remits within 6 months if the stressor is terminated; however, a persistent form of AD has been described if the stressor persists (First, 2013; Maercker and Lorenz, 2018). Furthermore, untreated AD poses the risk of persistent AD, and may pave the way for psychiatric disorders other than AD, particularly major depressive disorder and anxiety disorders (O’Donnell et al., 2016).

Epidemiological data are scarce since none of the major epidemiological studies included adjustment disorders among the conditions examined (Myers et al., 1984; Jenkins et al., 1997; Jacobi et al., 2004, 2014; Kessler et al., 2005). However, AD is reported to be common in primary care where rates of the disorder range from 1 to 18% (Casey et al., 1984; Blacker and Clare, 1988), and is also common in elderly persons as shown in a representative community survey (2.3%) (Maercker et al., 2008).

AD has been reported to be almost three times as common as major depression in acutely ill patients (13 versus 5%) (Silverstone, 1996). In potentially life-threatening diseases such as cancer, AD rates as high as 19.4% have been described, and AD has been observed in 15.4% of patients receiving palliative care (Mitchell et al., 2011). In up to one-third of breast cancer patients experiencing recurrence of their cancer, AD has been reported (Okano et al., 2001).

Pulmonary arterial hypertension (PAH) is a rare condition characterized by pulmonary vascular remodeling leading to right heart failure and death. Untreated, the estimated median survival of PAH was 2.8 years (D’Alonzo et al., 1991). Although treatment options have been improved during the last 20 years, PAH treatment is challenging: First, available treatments only reduce the progression of the disease course. Second, patients experience massive physical restrictions, leading to dyspnea, fatigue, exercise-induced syncope, suffocation, and edema, leading to decreased quality of life and decreased social functioning. Third, mortality is still high (3-year survival 70–80%) and for some patients lung transplantation remains the only treatment option. AD may pose further burdens on the patients, reducing their quality of life and psychological well-being (Hoeper et al., 2013; Galie et al., 2016).

Increased levels of anxiety and depression symptoms, and decreased quality of life have been observed in PAH, although AD has not been described so far (Larisch et al., 2014; Somaini et al., 2016). Divergent treatments such as cognitive behavioral therapy and low-intensity psychological interventions (self-help therapy, bibliotherapy, support groups, behavioral activation, mindfulness, meditation, relaxation, and e-mental health interventions) have been proposed for the treatment of AD, and three broader common components of these divergent strategies have been identified: (1) the enabling of individuals to reduce or remove the stressor, (2) interventions to improve coping with the stressor, and (3) stress reduction strategies (O’Donnell et al., 2018). However, to date there is only limited empirical evidence for these treatments in AD and a need for further studies and replication studies evaluating the efficacy of specific interventions in patients with AD have been proposed (O’Donnell et al., 2018). An alternative approach might be to take a more theory-driven perspective and modify the mechanisms that contribute to abnormal stress reactions. Research stimulated by the metacognitive model (Wells and Matthews, 1994) implicates metacognitive beliefs, worry and rumination in the maintenance and exacerbation of stress responses (e.g., Wells and Papageorgiou, 1995), and is supported by evidence that thought control strategies such as worry predict PTSD (e.g., Holeva et al., 2001). This psychological approach (MCT, Wells, 2009) has been found to be effective in both psychological and physical health contexts. The outcome of MCT in cardiac rehabilitation patients (Wells et al., 2018) is currently being evaluated, however, there is evidence of an association between metacognitive beliefs and psychological distress in other health conditions (e.g., Fisher et al., 2017). Overall, the therapy strategies used in MCT possibly prove a good fit to emotional distress in cardiac patients (McPhillips et al., 2018). We therefore examined if using MCT to treat a patient suffering from severe AD in the context of PAH was feasible and associated with symptom reduction.

## Case Presentation

### Biography

The patient described in this case report is a 34-year-old woman with the diagnosis of hereditary PAH. Several male family members on her father’s side had succumbed to the same disease around the age of 35 years. Her mother suffers from depression, and one brother has panic attacks. After finishing high school, the patient became a professional and worked in various physical health fields. She lives together with her boyfriend and in 2017 gave birth to her first child.

### Symptoms

The patient reported dyspnea on exertion after giving birth to a healthy child in 2017. However, despite this fact and the above-mentioned family history, diagnosis of PAH was not made until December 2018 when she was admitted to our hospital as an emergency with right heart failure after pulmonary infection. She recovered with supportive measures and introduction of PAH treatment with macitentan, an endothelin receptor antagonist, and tadalafil, a phosphodiesterase-5 inhibitor. When she returned home, she continued to experience severe limitations in everyday situations. For example, she was not able to carry her child as she felt too weak. In addition, she was afraid of any illness her child could infect her with. She felt incapable of looking after her child on her own and was dependent on other people’s support. As soon as she experienced signs of being ill, she went to specialists for check-ups. She was very quickly physically exhausted and had an increased need to sleep. Her situation led to intensive worrying about her self-image, her future, and her health. She cried more than before and experienced panic attacks several times per week. She feared her death and felt guilt toward her family members. Further on she repeatedly kept comparing her current situation to how it was before she was diagnosed, which led to despair and hopelessness. Her everyday life was dominated by anxiety, safety behaviors, and despair. She could hardly be by herself and was dependent on reassurance from others. She was grateful for the internal specialist’s referral to the department of psychiatry to seek help.

Consultation by the department of psychiatry resulted in the diagnosis of a severe adjustment disorder and she was registered for treatment.

### Assessment

In February 2019, she had a first appointment at the Department of Psychiatry, Social Psychiatry and Psychotherapy for a diagnostic assessment, and inpatient treatment started eventually in March. Treatment duration was 4 weeks. At both time points before treatment (T0a: diagnostic assessment, T0b: day of hospitalization) as well as at the end of treatment (T1) and 6 weeks after treatment ended (T2) she completed a set of questionnaires including the Hospital Anxiety and Depression Scale (Herrmann-Lingen et al., 2011), and the Metacognition Questionnaire (Wells and Cartwright-Hatton, 2004). At the start of treatment, she reported significant mental distress, indicated by a sum score of the anxiety subscale of the HADS of 13 points, and 7 points on the HADS depression subscale. These scores indicated that anxiety was severe and predominant. The scores of the MCQ-30 show that negative beliefs about uncontrollability and danger of worry were strongest. All scores are presented in Table 1. The patient gave written informed consent for the publication of this case report.

**Table 1:** Metacognitive beliefs (MCQ-30) and symptoms of anxiety/depression (HADS) according to self-rating scales, and walking distance over the course of MCT treatment and after 6-week follow-up.

	Pre (T0a)	Pre (T0b)	Post (T1)	Difference T0b − T1	FU (T2)	Difference T0b − T2
HADS (total)	20	20	9	−11 (55%)	1	−19 (95%)
Anxiety	13	13	6	−7 (54%)	0	−13 (100%)
Depression	7	7	3	−4 (57%)	1	−6 (86%)
MCQ-30 (total)	77	77	35	−42 (55%)	34	−43 (55%)
POS	17	16	8	−8 (50%)	6	−10 (63%)
NEG	21	22	8	−14 (64%)	10	−12 (55%)
CC	7	7	6	−1 (14%)	6	−1 (14%)
NC	15	14	6	−8 (57%)	6	−8 (57%)
CSC	17	18	7	−11 (61%)	6	−12 (67%)
Walking distance	351	358 m	439 m	+81 m (23%)	Not measured at T2 due to a cold	

*Initial HADS scores pointed to severe mental health problems according to AD. HADS scores for anxiety and depressive symptoms reduced to subthreshold levels after 4 sessions MCT, and even more declined after 6wk follow-up. Metacognitions also improved > 50% and remained stable at 6wk follow-up. Of note, physical capacity markedly improved by 23% measured by an increased walking distance. AD, adjustment disorder; PAH, pulmonary arterial hypertension; HADS, hospital anxiety and depression scale; MCQ-30, metacognitions questionnaire30; POS, positive beliefs about worry; NEG, negative beliefs about uncontrollability and danger of worry; CC, cognitive confidence; NC, need for control; CSC, cognitive self-consciousness*.

### Treatment

**Figure 1:**
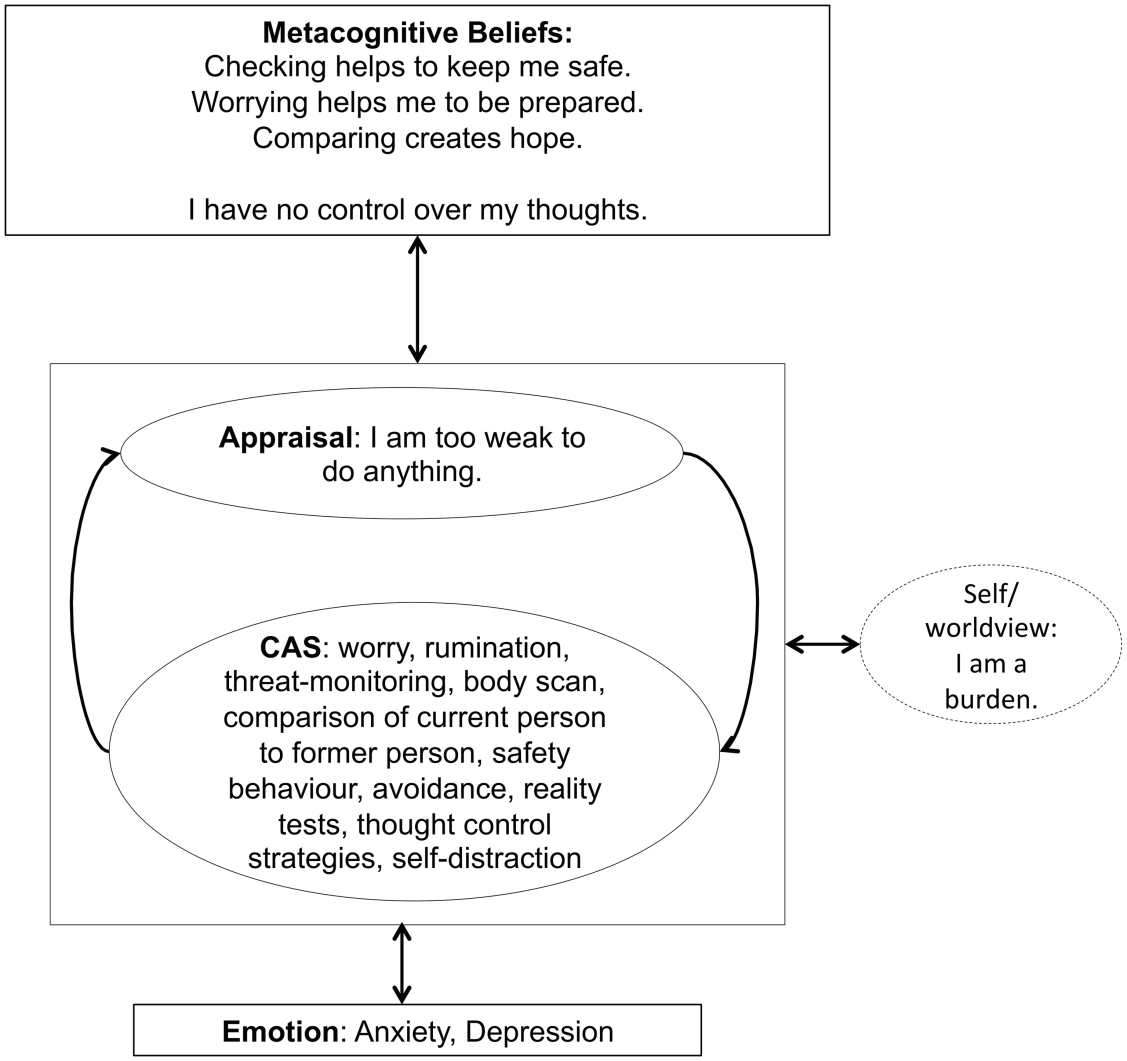
Individualized case formulation based on Wells generic model (Wells, 2009, p. 252) illustrating the maintenance of symptoms and underlying processes of the patient.

For the treatment with Metacognitive Therapy (MCT), the manual (Wells, 2009) was followed. During 4 weeks of inpatient treatment, the patient received weekly MCT sessions lasting 50 min each. In the first session, a personalized case formulation was developed using the generic model (Wells, 2009), which is presented in Figure 1. The patient was socialized to the model and the role of the cognitive attentional syndrome (CAS) was illustrated. Further, the patient was asked to rate the intensity of individual positive and negative metacognitive beliefs (Table 2). In the second session, Attention Training Technique (ATT; Wells, 1990) was introduced by using the German version of the audio file and the self-attention rating scale. Further, detached mindfulness was introduced by using the phone metaphor. For homework the patient was asked to do the ATT twice a day and practice worry postponement whenever her CAS was activated. In session number three, detached mindfulness was practiced again using the free association task several times. After the second repetition, subjectively difficult words were included in the task. In the beginning of the third session, the patient was also asked to rate the metacognitive beliefs formulated and rated in the first session (Table 2). The individual positive and negative metacognitive beliefs had already decreased to almost 0% and so no further challenging was undertaken. In the fourth session with the use of the “old plan – new plan” protocol was used to consolidate the change of strategies and attentional focus and metacognitive beliefs formulated in session 1 were again checked (Table 2). The patient was asked to repeat ATT after discharge for another 4 weeks.

**Table 2:** Rating of the patient’s individual metacognitive beliefs in each indicated MCT session.

Metacognitive beliefs	In session 1	In session 3	In session 4
**Positive metacognitive beliefs**			
Checking helps to keep me safe	100%	0%	0%
Worrying helps me to be prepared	100%	5%	5%
Comparing creates hope	90%	0%	0%
**Negative metacognitive beliefs**			
I have no control over my thoughts	95%	0%	0%

Assessment of metacognitive beliefs using the MCQ-30 (Table 1) demonstrated a significant reduction in positive and negative metacognitive beliefs, and a significant reduction in maladaptive coping strategies including all elements of the CAS. This improvement was accompanied by a reduction in symptoms of anxiety and depression assessed with the HADS (Table 1). Interestingly, we also found an improvement in physical symptoms. As part of the routine assessment for patients with PAH, walking distance is regularly measured, and the patient had a 23% increase in walking distance after the end of MCT treatment (Table 1). At 6 week follow-up, results concerning metacognitive beliefs and maladaptive coping strategies remained stable, while assessment of anxiety and depression symptoms revealed further improvement (Table 1).

It can be reported that the treatment was well tolerated by the patient and no adverse effects could be identified.

## Discussion

Our case report is notable in two ways: first, this is the first description of adjustment disorder as a consequence of a PAH diagnosis. Second, MCT was used for the first time to address AD in a PAH patient, without any further psychopharmacological medication. We chose this approach since according to the metacognitive theory, modifying the mechanisms that contribute to the development and maintenance of mental distress may improve AD.

Psychological distress has been associated with positive and negative metacognitive beliefs in a range of diseases including cancer (Thewes et al., 2013; Quattropani et al., 2017), Parkinson’s disease (Brown and Fernie, 2015), epilepsy (Fisher and Noble, 2017), chronic fatigue syndrome (Maher-Edwards et al., 2012), fibromyalgia (Kollmann et al., 2016), multiple sclerosis (Quattropani et al., 2018), and diabetes (Purewal and Fisher, 2018).

If the stress response is abnormal, meaning that it is out of proportion given the intensitiy of the stressor and followed by significant psychosocial impairment, AD can be diagnosed. AD is characterized by cognitive preoccupation with the disease itself, and its imagined consequences for one’s life and for significant others, resulting in emotional symptoms such as anxiety and depression, and in avoidance behaviors.

Psychotherapeutic and psychopharmacological treatment options for AD have recently been summarized in three review articles (Domhardt and Baumeister, 2018; O’Donnell et al., 2018; Stein, 2018). They found that the quality of evidence has been ranked low to very low (O’Donnell et al., 2018).

McPhillips et al. (2018) provide qualitative data on why MCT might be more effective than cognitive behavioral therapy (CBT) in the treatment of emotional distress in cardiac patients, although the validity of this hypothesis still needs to be shown. Still, a possible reason may be that content-related strategies like making a distinction between realistic and unrealistic thoughts may leave too much room for prolonged processing and may be ambiguous. A further aspect why the CBT model might be a poor fit is that patients describe diverse realistic negative automatic thoughts encompassing not only physical disease but also other areas of their lives (McPhillips et al., 2018). The perspective of MCT opens the opportunity to address emotional distress without analyzing the content of thoughts, which is often contradictory in AD. Perseverative thinking and underlying metacognitive beliefs can be targeted independently of realistic or unrealistic contents. A further advantage of MCT is its relatively short duration (Normann and Morina, 2018). In general, in CBT, more sessions are needed and the content related strategies used reach their limits in the treatment of AD.

In our patient, the metacognitions “Checking helps to keep me safe” or “Worrying helps me to be prepared” appeared to maintained dysfunctional coping strategies like threat monitoring, worrying, body scanning etc. and therefore preserved experiences of anxiety and insecurity. Over the course of treatment, the conviction in these metacognitions decreased. At the end of treatment, the patient reported new metacognitions like: “You can never be safe, so fighting for safety is useless” and “My body will tell me if it needs attention.” The change of metacognitions was accompanied by a decrease in both the anxiety and depression subscale of HADS. According to the metacognitive model, psychological disorders persist because of the effects of a state of thinking, the CAS, on emotional experiences and knowledge (Wells, 2009, p. 721). The CAS is controlled by positive and negative metacognitive beliefs. According to Wells (2009), this presents a range of possibilities for treatment that focus on removing the CAS, modifying metacognitive beliefs, and developing alternative ways of experiencing and relating to inner events (p. 729). In our case, the patient was introduced to the experience of being able to detach from her negative thoughts, reduce her CAS and apply her attention in a more flexible way. These experiences as well as the therapeutic style of addressing her concerns, e.g., with the use of the metacognitive socratic dialogue, lead to a change in metacognitive beliefs as indicated by the scores of the MCQ-30 and the ratings of her individual beliefs. Further modification of metacognitive beliefs was associated with a reduction of clinical symptoms indicated by a decrease of the HADS scores.

An interesting finding belongs to the improvement in physical parameters, i.e., greater walking distance. One can interpret this finding in two ways, either “psychological” as improved confidence of the patient in her physical capacity, or “somatic” as improved physical functioning once the psychological distress was reduced.

Our patient tolerated the intervention and gave positive feedback that she felt well understood and received what she needed. According to McPhillips et al. (2019), the psychological needs of cardiac rehabilitation patients can include the wish not to disclose their concerns. Therefore content-focused therapy like CBT may not be tolerated by these patients. In such cases MCT which is process-focused and allows patients to keep the content of thoughts private may have greater acceptance than other interventions.

Psychopharmacological treatment has also been discussed in AD. Treatment options include the use of benzodiazepines (alprazolam, diazepam, clorazepat, lormetazepam), antidepressants (mianserin, tianeptine, trazodone, viloxazine), plant extracts/herbals (euphytose, Ginko-Biloba, Kava-Kava), anxiolytics (etifoxine), 5-HT1A agonists (buspiron), and neutraceuticals (s-adenosylmethionine). However, only 11 randomized-controlled trials including 1,195 AD patients have been documented, yielding in part contradictory results (Stein, 2018). Furthermore, psychopharmacological treatment may be limited by drug-induced side effects, including pharmacokinetic and pharmacodynamics alterations such as drug–drug interactions, induction/inhibition of the cytochrome-P-450 system, or indirect drug effects that all may interact with the drugs necessary for treating the underlying disease (Huang et al., 2008; Kahl, 2018; Kahl et al., 2019). This particularly applies to patients with cardiorespiratory impairment (Kahl et al., 2017, 2018). Therefore, non-pharmacological treatments may be favored in patients who develop AD in the context of an underlying physical illness.

### Limitations

As this was an important inpatient treatment, other aspects of being on a psychiatric ward may have influenced the outcome. We rate this factor as low as she was the only patient with AD within the group of patients and was excluded from other forms of psychological interventions. A further limitation can be seen in the lack of a direct evaluation of the therapy process. Further, in the absence of a control condition we cannot rule out a placebo or a therapist effect. Unfortunately, we did not explicitly measure the CAS with the use of a questionnaire. However, according to the metacognitive model, we expect that changing metacognitive beliefs should lead to reduced CAS activity. In accordance with this, decreased worry intensity and less threat monitoring were reported by the patient. Another indicator for reduced CAS activity may be seen in reduced clinical symptoms. In future, a larger sample explored *via* single case or trial methodology is needed to investigate the use and effectiveness of MCT in the treatment of AD in the context of an underlying physical disorder.

## Conclusion

We conclude that MCT might be promising for patients with AD with an underlying physical disorder. Future studies examining acute and sustained effects of MCT in patients with AD in the context of different physical disorders are warranted.

## Data Availability Statement

The datasets generated for this study are available on request to the corresponding author.

## Ethics Statement

The studies involving human participants were reviewed and approved by Ethics Committee Hannover Medical School. The patients/participants provided their written informed consent to participate in this study. Written informed consent was obtained from the individual(s) for the publication of any potentially identifiable images or data included in this article.

## Author Contributions

LW was in charge of the therapy plan, was the therapist treating the patient, and wrote the manuscript. FN served as a co-therapist and also carried out the psychological assessments. KO and MH were the attending physicians for any somatic concern. Further on they provided training for the other authors on PAH and wrote parts of the manuscript. JF served as a co-physician and carried out the somatic assessments. KK supervised the treatment and wrote the manuscript.

### Conflict of Interest

The authors declare that the research was conducted in the absence of any commercial or financial relationships that could be construed as a potential conflict of interest.
